# A personal memory aid augmenting the ‘My five moments for hand hygiene’ concept

**DOI:** 10.1186/1753-6561-5-S6-P28

**Published:** 2011-06-29

**Authors:** HS Sax

**Affiliations:** 1 Infection control program, University of Geneva Hosptials and Medical Faculty, Geneva, Switzerland

## Introduction / objectives

Human memory capacity is limited and even relatively simple concepts are not easily retained if not central to the individual's emotional engagement. The 'My five moments for hand hygiene' (Sax H, et al. J Hosp Infect 2007;67:9-21) has been designed to be simple and 'sticky'. But even so, as informal surveys show, only 2 of 5 hand hygiene indications are accessible in active memory and correctly reproduced by healthcare workers upon request.

## Methods

Search for a simple memory aid using concepts of emotional design and human factors engineering principles through a heuristic try and error design process.

## Results

The solution is simple, almost for-free, and in most basic healthcare settings universally available. It enhances memorizing five distinct indications for hand hygiene and correct use of disposable gloves through a phase of personal physical challenge, joy of final success, simplicity and the principal of chunking the 6 items (5 indications for hand hygiene and correct glove use) into 3 items. Preliminary small-scale testing shows a strong memory-enhancing effect.

## Conclusion

This invention has a lifesaving potential at near zero cost. Very simple design solutions according to human factors principles can be very effective.

## Disclosure of interest

None declared.

